# Association between estimated pulse wave velocity and impaired fasting glucose: a multicenter retrospective cohort study in China

**DOI:** 10.3389/fendo.2026.1763973

**Published:** 2026-04-27

**Authors:** Jintao Jiang, Renzhe Lin, Juan Wu, Sen Li, Zitian Luo, Huankai Zhang, Longsheng Zhang, Duo Yang

**Affiliations:** 1 Department of Gastroenterology, Jieyang People’s Hospital, Jieyang, Guangdong, China; 2 First Clinical Medical College, Guangdong Medical University, Zhanjiang, Guangdong, China; 3 Department of Anesthesiology, Jieyang People’s Hospital, Jieyang, Guangdong, China; 4 Department of Clinical Laboratory, Jieyang People’s Hospital, Jieyang, Guangdong, China

**Keywords:** diabetes mellitus, endocrinology, estimated pulse wave velocity, fasting plasma glucose, impaired fasting glucose, predictive markers

## Abstract

**Background:**

Prediabetes, primarily impaired fasting glucose (IFG), represents a critical window for preventing diabetes and its vascular sequelae. Estimated pulse wave velocity (ePWV) is a noninvasive marker of aortic stiffness and a predictor of cardiovascular events. Although arterial stiffness is linked to early glucose metabolism abnormalities, the independent association of ePWV with incident IFG remains unclear. This study aimed to elucidate this relationship in a multi-province Chinese cohort and to identify potential risk thresholds.

**Methods:**

We performed a retrospective analysis of 184,291 Chinese adults with normal baseline fasting plasma glucose from the Rich Healthcare Group. Participants were stratified into four quartiles according to their ePWV values. Kaplan-Meier (K-M) survival analysis and Cox proportional hazards regression models were subsequently employed to evaluate the relationship between ePWV and the incidence of IFG. Restricted cubic spline (RCS) analysis was employed to explore potential non-linear relationships and identify inflection points. To evaluate the robustness of the results, sensitivity and subgroup analyses were performed.

**Results:**

During a median follow-up period of 3.0 years, 11.28% of participants (n=20,783) developed incident IFG. Following multivariable adjustment for gender, BMI, FPG, blood lipids, liver and renal function, and behavioral factors, each 1 m/s increase in ePWV was associated with a hazard ratio (HR) of 1.19 (95% CI: 1.18-1.2) for developing IFG. Furthermore, K-M survival analysis revealed that the incidence of IFG demonstrated a progressive rise in tandem with increasing levels of ePWV quartiles. RCS revealed a nonlinear association was observed, characterized by a threshold was identified at 8.365 m/s, below which the relationship exhibited a distinct change in trend, each 1 m/s rise conferred a 33% higher risk. The positive association between elevated ePWV and IFG risk remained robust across all subgroups examined.

**Conclusions:**

In this large retrospective cohort study, elevated ePWV was independently associated with an increased risk of incident IFG, exhibiting a nonlinear relationship with a more pronounced risk gradient below 8.365 m/s. These findings suggest ePWV may serve as a simple, non-invasive marker for early identification of individuals at high risk for IFG.

## Background

Diabetes mellitus (DM) and its prediabetic antecedents constitute a prevalent cluster of chronic metabolic disorders that impose a major global public-health burden (1). According to the latest data, in 2024, one in nine adults worldwide was living with diabetes. The number of adults with diabetes exceeded 500 million and is projected to rise to close to 900 million by 2050 (2). Prediabetes (Pre-DM), a condition characterized by either impaired fasting glucose (IFG) or impaired glucose tolerance (IGT), constitutes a pivotal intermediate phase in the natural history of DM (3). Individuals with isolated IFG face a substantial risk of longitudinal progression, with approximately 25% developing overt DM within 3–5 years (4, 5). According to authoritative literature, impaired glucose tolerance (IGT) affected approximately 634.8 million adults globally in 2024, accounting for 12.0% of the world’s adult population, while IFG affected approximately 487.7 million adults, representing 9.2% of the global adult population. It is projected that by 2050, the number of individuals with IGT will rise to 846.5 million (constituting 12.9% of the global adult population), and those with IFG will increase to 647.5 million (9.8%). Furthermore, from 2021 to 2024, the prevalence of IGT increased from 9.1% to 12.0%, and that of IFG rose from 5.8% to 9.2%, indicating a marked upward trend in the global prevalence of pre-DM (6).

In the absence of timely intervention, individuals with pre-DM progress rapidly to overt DM and become concurrently exposed to the full spectrum of diabetic complications. Both pre-DM and established DM substantially elevate the likelihood of atherosclerotic cardiovascular disease, chronic kidney disease, retinopathy, and site-specific malignancies, and they are independently associated with accelerated cognitive decline, major depressive disorder, and all-cause mortality, thereby imposing a formidable socioeconomic burden (7, 8). However, because IFG is a potentially reversible entity, early identification and prompt intervention can restore normoglycaemia and, consequently, attenuate the subsequent incidence of DM and major adverse cardiovascular events. Consequently, the discovery of simple, reproducible, and pathophysiologically informative early-warning biomarkers has become a cardinal priority in contemporary DM-prevention strategies (9). Established risk factors for pre-DM include advanced age, general and central adiposity, physical inactivity, arterial hypertension, dyslipidaemia, and a positive family history of DM (10–12). However, these conventional risk factors exhibit only modest predictive accuracy for incident IFG, whereas reference procedures such as insulin sensitivity indices are costly, technically demanding, and poorly suited to large-scale screening. Consequently, there is a pressing requirement to create straightforward and resilient early biomarkers that can be deployed in routine practice. Timely intervention guided by such early biomarkers has been proven to substantially reduce the incidence of DM, underscoring its profound public-health importance. Conventional screening relies on the determination of fasting plasma glucose (FPG), the measurement of blood glucose concentrations, or the performance of an oral glucose tolerance test (OGTT) and constitutes a standard diagnostic approach and diagnostic procedures. However, the former is susceptible to acute perturbations, whereas the latter is limited by suboptimal patient adherence. Emerging evidence indicates that vascular functional indices begin to deteriorate during the earliest stages of glucoregulatory impairment, thereby offering prodromal warning capability. Estimated pulse wave velocity (ePWV) is a calculated surrogate measure of arterial stiffness, derived from an individual’s age and brachial blood pressure (BP) using validated equations, rather than a direct physical measurement (13, 14). This parameter exhibits marked alignment with carotid-femoral pulse wave velocity, yet it requires no specialized instrumentation and is therefore well suited to large-scale epidemiological investigations. An elevated ePWV signifies increased large-artery stiffness, reflecting fragmentation of elastic lamellae, collagen deposition, and smooth-muscle hypertrophy that collectively reduce buffering of pulsatile pressure and precipitate heightened cardiac afterload and microcirculatory injury (15). Evidence demonstrates that ePWV constitutes an independent predictor of incident cardiovascular incidents and all-cause mortality (16).

Arterial stiffness contributes to dysglycemia through interconnected pathophysiological pathways.​ Elevated aortic stiffness, indicated by a higher ePWV, increases central pulse pressure and disturbs endothelial shear stress. This reduces nitric oxide bioavailability, impairing endothelium-dependent vasodilation and leading to capillary rarefaction and underperfusion in skeletal muscle—key mechanisms that compromise insulin-mediated glucose uptake and promote insulin resistance (17). Concurrently, vascular remodeling is associated with chronic low-grade inflammation and oxidative stress; pro-inflammatory cytokines disrupt insulin signaling, while reactive oxygen species can impair pancreatic β-cell function (18). Moreover, early hyperglycemia promotes advanced glycation end-product (AGE) formation, which further exacerbates arterial stiffness through collagen cross-linking and inflammatory activation, establishing a vicious cycle (19).

A newly published Chinese cohort investigation recognized ePWV as a determinant of incident DM (20). Given that DM evolves progressively from antecedent states such as IFG and that microvascular dysfunction and endothelium-dependent vasodilatory impairment are already present during IFG, it is plausible that arterial stiffness contributes to the pathogenesis of IFG. Notably, mean arterial pressure, a component of the ePWV equation, has been associated with IFG risk. In addition, both pulse pressure (PP) and the pulse pressure index (PPI) have been linked toward the likelihood of progressing to incident IFG. However, the predictive utility of ePWV itself for IFG onset requires direct evaluation. Therefore, leveraging a retrospective multicentre cohort spanning multiple provinces in China, this investigation seeks to elucidate the independent relationship linking ePWV to incident IFG, thereby providing evidence for low-cost, individualized pre-DM screening and early intervention at the primary-care level and promoting the clinical integration of vascular functional indices in the primordial prevention of metabolic disease.

## Method

### Data source

The raw data utilized in this study are publicly available from the Dryad Digital Repository. The dataset originates from an academic study published by Chen et al. in 2018 (21), and the complete data files can be accessed by researchers via the link (https://doi.org/10.5061/dryad.ft8750v). The dataset employed in the present study was collected retrospectively from a nationwide clinical database curated by the Rich Healthcare Group throughout China. This archive includes clinical data collected between 2010 and 2016 from participants receiving routine health check-ups. The initial research protocol received formal approval from the Ethics Review Board of the Rich Healthcare Group. In line with Dryad’s data stewardship policy, third-party investigators are permitted to perform non-commercial academic analyses provided that the terms stipulated by the original authors are observed.

### Study population

In the original study, Chen and colleagues investigated the association of body mass index (BMI) and age with the onset of DM in a cohort of 211,833 adult participants who underwent at least two health assessments conducted between 2010 and 2016. Importantly, they found that elevated BMI was independently associated with an increased risk of incident DM, a relationship that was particularly pronounced in younger adults. This comprehensive dataset provides detailed baseline clinical characteristics, including demographic profiles, BP, and FPG.

**Figure 1:**
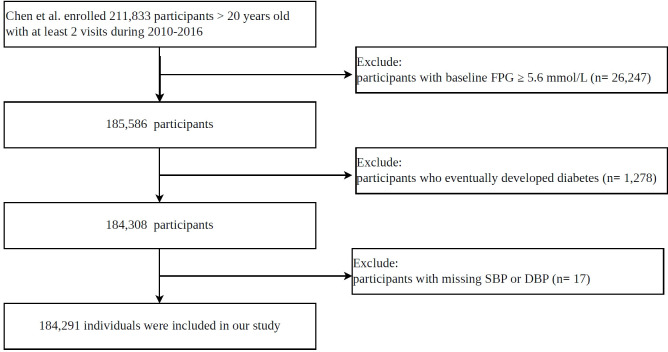
Flowchart of the study.

While the primary study focused on the eventual onset of DM, in the present secondary analysis, we restricted the cohort to participants whose baseline FPG was within the normal range to systematically evaluate the effect of the initial ePWV on the development of incident IFG. To align with this objective, we sequentially excluded individuals whose baseline FPG exceeded 5.6 mmol/L, who progressed to overt DM during follow-up, or whose systolic or diastolic BP values were missing. This screening process left 184,291 eligible participants. **Figure 1** illustrates the complete participant enrollment and screening process.

### Definitions of the exposure and outcome variable

In this study, ePWV served as the exposure variable. Mean arterial pressure (MAP) was computed as (1/3) × SBP + (2/3) × DBP. The ePWV was then calculated using the following validated equation (14, 22): ePWV= 9.587 -0.402 ×Age + 0.00456 × Age^2^ - 0.00002621 × Age^2^ × MAP + 0.003176 × Age × MAP - 0.01832 × MAP. The primary endpoint was incident IFG, defined according to the American Diabetes Association criteria as an FPG concentration between 5.6 and 6.9 mmol/L in individuals without previously diagnosed DM. The duration of follow-up was characterized as the time span between the initial and final observation dates of the available health examination.

### Covariates

The variables analyzed in this study were classified into five distinct categories: (1) Demographics: age and gender; (2) Body measurements: height, body weight, and BP; (3) Laboratory parameters: The measured parameters included FPG, total cholesterol (TC), triglycerides (TG), low-density lipoprotein cholesterol (LDL-C), high-density lipoprotein cholesterol (HDL-C), alanine aminotransferase (ALT), aspartate aminotransferase (AST), blood urea nitrogen (BUN), and serum creatinine (Scr); (4) Behavioral factors: smoking and drinking status; (5) Family history of DM. The collection of data encompassed demographic characteristics, lifestyle factors, and family history with a standardized, structured questionnaire that was administered. Measurements of height, BP, and body weight were analyzed and obtained by certified staff who had completed centralized training and passed competency tests. BMI was computed using body weight in kilograms divided by the square of height in meters. Smoking and drinking statuses were classified in the original database as current, ever, or never. Venous blood was drawn following a minimum 10-hour nocturnal fast, and all biochemical indices were determined with a Beckman Coulter AU5800 automatic analyzer.

### Missing data processing

In observational studies, incomplete data are unavoidable. In this study, 10 variables contained missing data, comprising 8 continuous and 2 categorical variables. The proportion of missing the corresponding specific values for each variable are detailed in **Supplementary Table S1**. To minimise bias, missing biochemical data, including ALT, AST, TG, TC, LDL-C, HDL-C, BUN, and Scr, were imputed using multiple imputation. The imputation process was conducted using the multiple imputation by chained equations (MICE) method via the mice package in the R environment (23), with five iterations to generate five complete datasets. For smoking and drinking status, individuals with missing information were retained as a separate “Not recorded” category. We analyzed both the multiply imputed dataset and a complete-case dataset that excluded participants with missing covariates, with the complete-case dataset results reported in the **Supplementary Material**.

### Statistical method

**Figure 2:**
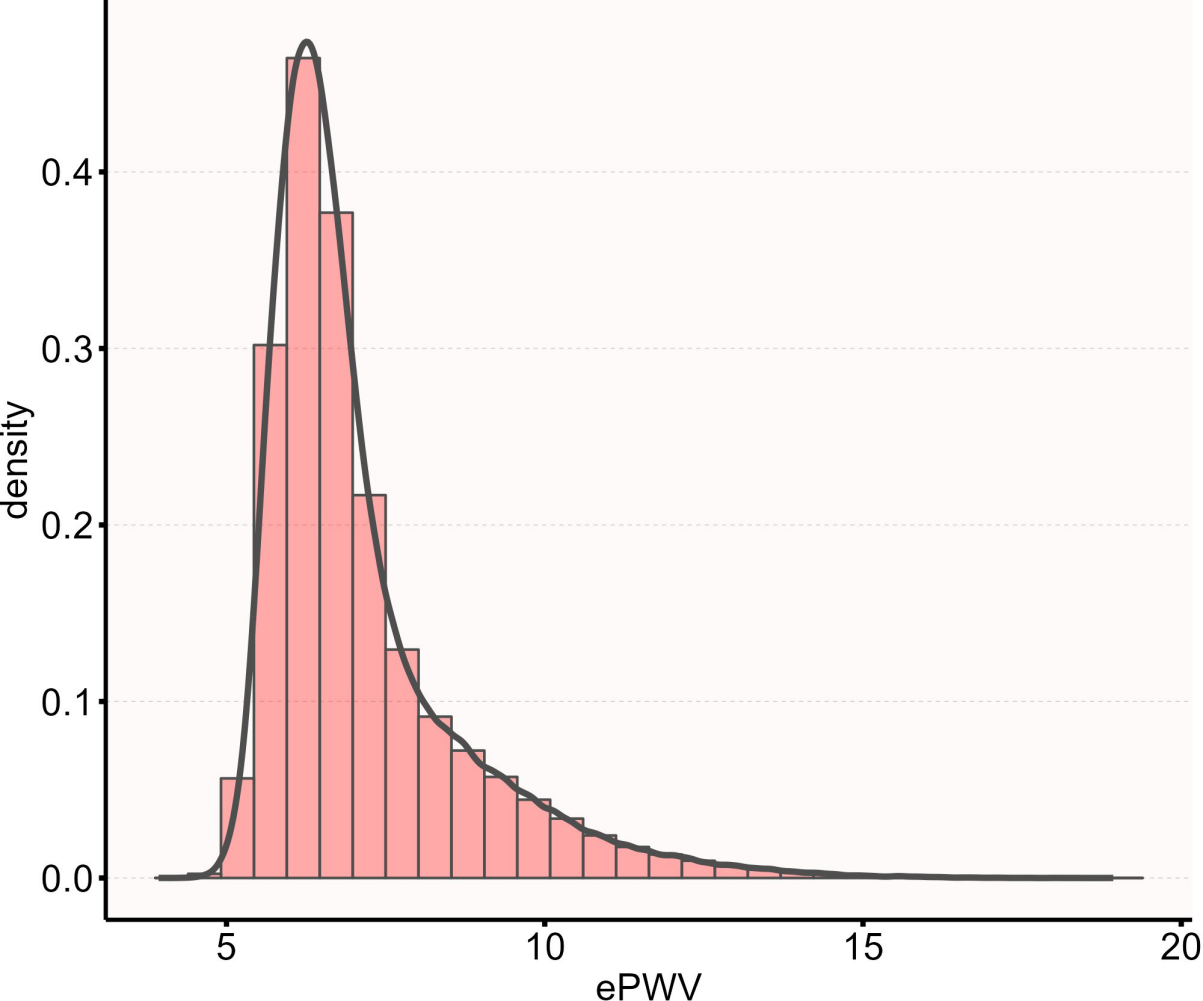
The distribution of ePWV.

The calculated ePWV in this study was approximately normally distributed. The distribution of ePWV in the study population is shown in **Figure 2**. To facilitate clinical interpretation and to examine the potential non-linear association between ePWV and IFG risk, participants were stratified into four groups (Q1 to Q4) based on the quartiles of their baseline ePWV values. This categorization allows for the comparison of risk gradients across different levels of arterial stiffness and is a commonly used approach to evaluate dose-response relationships in epidemiological studies. Based on the quartiles of ePWV, all participants were divided into four groups: Q1 (n = 46073, ePWV ≤ 6.092 m/s), Q2 (n = 46049, ePWV ≤ 6.632 m/s), Q3 (n = 46088, ePWV ≤ 7.602 m/s), and Q4 (n = 46081, ePWV ≤ 18.932 m/s). The Kolmogorov-Smirnov test was employed to assess the normality of continuous variables. Data following a normal distribution were presented as mean ± standard deviation, while those deviating from normality were expressed as median with interquartile range [M(Q1, Q3)]. Categorical variables were displayed as frequencies (n) alongside percentages (%). Inter-group comparisons for normally distributed continuous variables were conducted with one-way analysis of variance. Continuous variables violating the normality assumption were examined using the Kruskal-Wallis H test for multi-group comparisons. The chi-square test was applied to assess associations between categorical variables.

The incidence of IFG was displayed using Kaplan-Meier (K-M) curves were constructed based on ePWV quartiles, and the log-rank test was employed to evaluate survival disparities among the four groups. To investigate the association between ePWV and the risk of incident IFG, ePWV was categorized into quartiles. Multivariable cox proportional hazards regression models were used to compute HRs and their associated 95% confidence intervals incident IFG, with adjustment for potential confounders. Model I comprised gender, BMI and baseline FPG, model II further incorporated the following baseline covariates: TC, TG, HDL-C, LDL-C, ALT, AST, BUN, Scr, smoking status, drinking staus, and family history of DM. Given that the exposure variable, ePWV, is mathematically derived from age and blood pressure, these components were not included as separate covariates in the multivariable Cox regression models to avoid structural multicollinearity. We assessed the variance inflation factor (VIF) for all other covariates included in Model II. All VIF values were below 5, indicating no substantial multicollinearity among the adjusted variables. To examine a possible nonlinear relation between ePWV and incident IFG, we fitted restricted cubic spline models to produce smoothed exposure–response curves. Upon detection of a nonlinear relationship, a two-piecewise regression model was employed to explore the potential threshold effect of ePWV on the risk of newly developed IFG. To evaluate the association between ePWV and incident IFG risk across different population strata, we conducted stratified Cox proportional hazards regression analyses. Subgroups were defined by age, sex, BMI, smoking status, drinking status, family history of diabetes, blood pressure, and lipid profiles. In each stratified model, we adjusted for a consistent set of covariates, including BMI, baseline fasting plasma glucose, ALT, AST, TG, TC, LDL−C, HDL−C, BUN, Scr, smoking status, drinking status, and family history of diabetes, while the stratification variable for that specific analysis was excluded from the adjustment set.

### Statistical software

All statistical analyses were conducted with R software (version 4.2.2; obtainable from http://www.R-project.org) and the Free Statistics Analysis Platform (version 1.9.2; retrievable at http://www.clinicalscientists.cn/freestatistics). *P*-values below 0.05 on a two-tailed basis were considered to indicate statistical significance. As the present study employed pre-existing data, sample size estimation was not undertaken.

## Results

### Baseline demographic and clinical profiles

**Figure 3:**
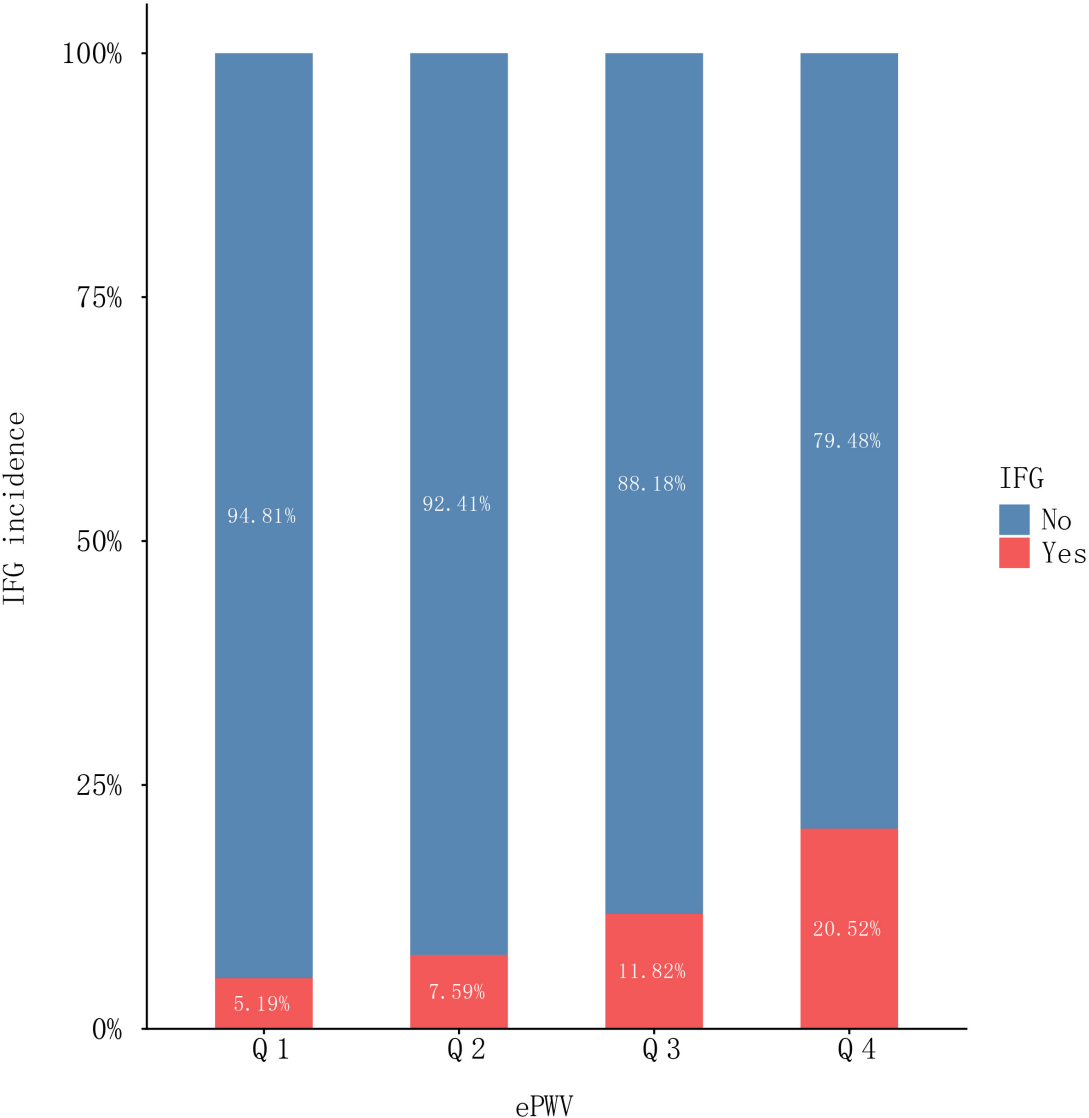
Stacked column chart of IFG incidence. The horizontal axis displays the ePWV quartiles (Q1, Q2, Q3, Q4) and the vertical axis shows the percentage incidence of IFG. Red segments represent incident cases, blue segments represent non-incident cases, and the exact percentages for each category are labeled within the columns.

**Table 1** presents an overview of the baseline demographic and clinical profiles of the study participants cohort, comprising 184,291 individuals. The average age of the study cohort was 41.02 ± 12.10 years, with males constituting 97,809 participants, or 53.07% of the total. During a median follow-up duration of 3.0 years, a cumulative count of 20,783 participants (11.28%) were documented to have progressed to IFG. As shown in **Figure 3**, the incidence of IFG showed a marked increase across ePWV quartiles, rising from 5.19% in Q1 to 20.52% in Q4, representing an approximately fourfold absolute increase in incidence​ from the lowest to the highest ePWV category (*P* < 0.001).

**Table 1:** Baseline demographic and clinical characteristics of the study cohort, stratified by ePWV quartiles.

Variables	Total (n =184291)	ePWV				
		Q1 (n = 46073)	Q2 (n = 46049)	Q3 (n = 46088)	Q4 (n = 46081)	*P*-Value
ePWV (m/s), Mean ± SD	7.11 ± 1.57	5.75 ± 0.25	6.36 ± 0.16	7.03 ± 0.27	9.32 ± 1.55	< 0.001
Age (years), Mean ± SD	41.02 ± 12.10	33.48 ± 5.05	34.62 ± 6.25	39.23 ± 7.88	56.76 ± 10.48	< 0.001
Gender, n (%)						< 0.001
Male	97809 (53.07)	14820 (32.17)	25345 (55.04)	29896 (64.87)	27748 (60.22)	
Female	86482 (46.93)	31253 (67.83)	20704 (44.96)	16192 (35.13)	18333 (39.78)	
Height (cm), Mean ± SD	166.39 ± 8.32	164.66 ± 7.95	167.43 ± 8.32	168.15 ± 8.23	165.33 ± 8.28	< 0.001
Body weight (kg), Mean ± SD	63.97 ± 12.04	58.19 ± 10.18	63.46 ± 11.75	67.41 ± 12.44	66.82 ± 11.40	< 0.001
BMI (kg/m^2^), Mean ± SD	22.99 ± 3.27	21.37 ± 2.71	22.52 ± 3.10	23.72 ± 3.31	24.35 ± 3.08	< 0.001
SBP (mmHg), Mean ± SD	117.83 ± 15.81	102.59 ± 8.17	114.67 ± 9.11	122.46 ± 11.60	131.59 ± 16.20	< 0.001
DBP (mmHg), Mean ± SD	73.53 ± 10.60	62.57 ± 4.96	71.12 ± 4.97	77.88 ± 7.38	82.54 ± 10.94	< 0.001
MAP (mmHg), Mean ± SD	88.30 ± 11.40	75.91 ± 4.70	85.64 ± 4.60	92.74 ± 7.51	98.89 ± 11.30	< 0.001
Baseline FPG (mmol/L), Mean ± SD	4.77 ± 0.49	4.67 ± 0.49	4.74 ± 0.48	4.79 ± 0.49	4.87 ± 0.47	< 0.001
TC (mmol/L), Mean ± SD	4.67 ± 0.88	4.40 ± 0.79	4.51 ± 0.82	4.70 ± 0.86	5.04 ± 0.92	< 0.001
TG (mmol/L), M (IQR)	1.02 (0.71, 1.53)	0.80 (0.60, 1.10)	0.94 (0.68, 1.37)	1.12 (0.79, 1.69)	1.34 (0.94, 1.94)	< 0.001
HDL-C (mmol/L), Mean ± SD	1.32 ± 0.30	1.36 ± 0.30	1.31 ± 0.30	1.29 ± 0.30	1.32 ± 0.31	< 0.001
LDL-C (mmol/L), Mean ± SD	2.72 ± 0.67	2.54 ± 0.61	2.62 ± 0.63	2.75 ± 0.66	2.97 ± 0.71	< 0.001
ALT (U/L), M (IQR)	17.50 (12.60, 26.60)	14.00 (10.70, 20.00)	17.00 (12.00, 26.00)	20.10 (14.00, 31.90)	20.00 (14.90, 28.30)	< 0.001
AST (U/L), M (IQR)	23.00 (19.40, 27.60)	21.00 (18.10, 25.00)	22.00 (19.00, 26.60)	23.30 (20.00, 28.50)	25.00 (21.20, 29.80)	< 0.001
BUN (mmol/L), Mean ± SD	4.61 ± 1.17	4.34 ± 1.09	4.50 ± 1.12	4.64 ± 1.14	4.95 ± 1.24	< 0.001
Scr (μmol/L), Mean ± SD	69.59 ± 15.71	64.54 ± 14.16	69.67 ± 14.99	71.86 ± 15.19	72.29 ± 17.12	< 0.001
Smoking status, n (%)						< 0.001
Current smoker	9672 (5.25)	1204 (2.61)	2104 (4.57)	2727 (5.92)	3637 (7.89)	
Ever smoker	2120 (1.15)	353 (0.77)	612 (1.33)	701 (1.52)	454 (0.99)	
Never smoker	39220 (21.28)	10311 (22.38)	10779 (23.41)	10355 (22.47)	7775 (16.87)	
Not recorded	133279 (72.32)	34205 (74.24)	32554 (70.69)	32305 (70.09)	34215 (74.25)	
Drinking status, n (%)						< 0.001
Current drinker	988 (0.54)	83 (0.18)	156 (0.34)	249 (0.54)	500 (1.09)	
Ever drinker	7370 (4.00)	1248 (2.71)	1997 (4.34)	2440 (5.29)	1685 (3.66)	
Never drinker	42654 (23.14)	10537 (22.87)	11342 (24.63)	11094 (24.07)	9681 (21.01)	
Not recorded	133279 (72.32)	34205 (74.24)	32554 (70.69)	32305 (70.09)	34215 (74.25)	
Family history of DM, n (%)						< 0.001
No	180644 (98.02)	45094 (97.88)	45133 (98.01)	45033 (97.71)	45384 (98.49)	
Yes	3647 (1.98)	979 (2.12)	916 (1.99)	1055 (2.29)	697 (1.51)	
IFG, n (%)						< 0.001
No	163508 (88.72)	43684 (94.81)	42556 (92.41)	40641 (88.18)	36627 (79.48)	
Yes	20783 (11.28)	2389 (5.19)	3493 (7.59)	5447 (11.82)	9454 (20.52)	

Across increasing quartiles of ePWV (Q1 to Q4), the values of age, SBP, DBP, MAP, BMI, baseline FPG, TC, TG, LDL-C, BUN, Scr, and AST exhibited a progressive increase. ALT levels progressively increased from Q1 to Q3 but slightly decreased in Q4. HDL-C levels were significantly different across quartiles (*P* < 0.001), with lower mean values observed in Q2 and Q3 compared to Q1 and Q4. The percentage of male participants rose from 32.17% in Q1 to a peak of 64.87% in Q3, before slightly decreasing to 60.22% in Q4. Current smoking and current drinking became more prevalent with higher ePWV values, whereas the shares of never smokers and never drinkers peaked in Q2 and subsequently declined in the higher quartiles (*P* < 0.001 for each). The proportion of participants with a positive family history of DM varied from 1.51% to 2.29% among quartiles (*P* < 0.001).

### Association between the ePWV and the IFG events

**Figure 4:**
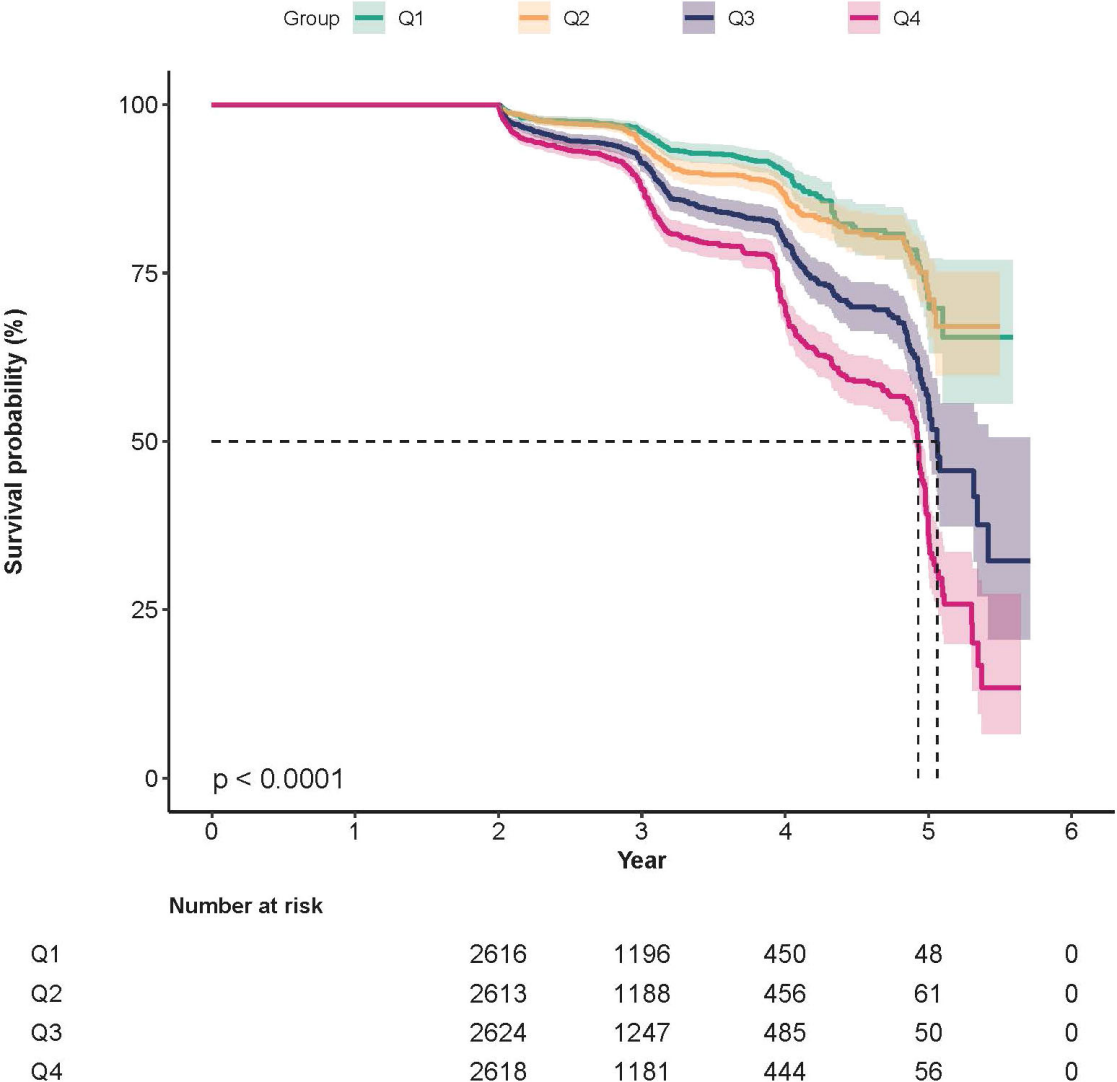
K-M curves illustrate IFG incidence by ePWV quartiles (Log-rank test *P* < 0.0001).

The results of the Cox proportional hazards regression are shown in **Table 2**. In the unadjusted analysis, each 1 m/s rise in ePWV was linked to a HR of 1.28 (95%CI = 1.27 – 1.29, *P*< 0.001) for the risk of incident IFG. Compared with Q1, the HRs (95%CI) for Q2, Q3, and Q4 were 1.42 (1.35 – 1.50), 2.08 (1.98 – 2.19), and 3.78 (3.62 – 3.96), respectively, with a trend *P* < 0.001. Following adjustment after adjusting for gender, BMI, and baseline FPG in Model I, each 1 m/s rise in ePWV corresponded to a HR of 1.19 (95%CI = 1.18 – 1.20, *P* < 0.001). Versus Q1, the HRs (95% CI) for Q2, Q3, and Q4 were 1.14 (1.08 – 1.21), 1.42 (1.35 – 1.49), and 2.21 (2.11 – 2.32), respectively, and the trend test yielded *P* < 0.001. In Model II, each 1 m/s increase in ePWV was associated with a 19% higher risk of incident IFG (HR = 1.19, 95% CI: 1.18 – 1.20). When analyzed by quartiles, a clear gradient of increasing risk​ was observed with higher ePWV levels. Compared to participants in Q1, the risk of IFG was 1.14- fold in Q2 (HR = 1.14, 95% CI: 1.08 – 1.20), 1.41-fold in Q3 (HR = 1.41, 95% CI: 1.34 – 1.48), and 2.2- fold in the Q4 (HR = 2.20, 95% CI: 2.09 – 2.31). K-M curves (**Figure 4**) demonstrated a stepwise increase in the cumulative incidence of IFG across ascending ePWV quartiles, with successively higher incidences in Q1, Q2, Q3 and Q4 (Log-rank test *P* < 0.001).

**Table 2:** The association between ePWV and the risk of IFG among the study population in different models.

Variables	Crude model		Model I		Model II	
	HR (95%CI)	*P*-Value	HR (95%CI)	*P*-Value	HR (95%CI)	*P*-Value
ePWV, m/s	1.28 (1.27, 1.29)	< 0.001	1.19 (1.18, 1.20)	< 0.001	1.19 (1.19, 1.20)	< 0.001
(ePWV quartiles)						
Q1	1.00 (Reference)		1.00 (Reference)		1.00 (Reference)	
Q2	1.42 (1.35, 1.50)	< 0.001	1.14 (1.08, 1.21)	< 0.001	1.14 (1.08, 1.20)	< 0.001
Q3	2.08 (1.98, 2.19)	< 0.001	1.42 (1.35, 1.49)	< 0.001	1.41 (1.34, 1.48)	< 0.001
Q4	3.78 (3.62, 3.96)	< 0.001	2.21 (2.11, 2.32)	< 0.001	2.20 (2.09, 2.31)	< 0.001
*P* for trend		< 0.001		< 0.001		< 0.001

Crude model, no additional covariates were adjusted in our analysis.

Model I, adjusted for gender, BMI, and baseline FPG.

Model II, further adjusted for ALT, AST, TG, TC, LDL-C, HDL-C, BUN, Scr, smoking status, drinking status, and family history of DM.

### The nonlinear relationship between ePWV and IFG

**Figure 5:**
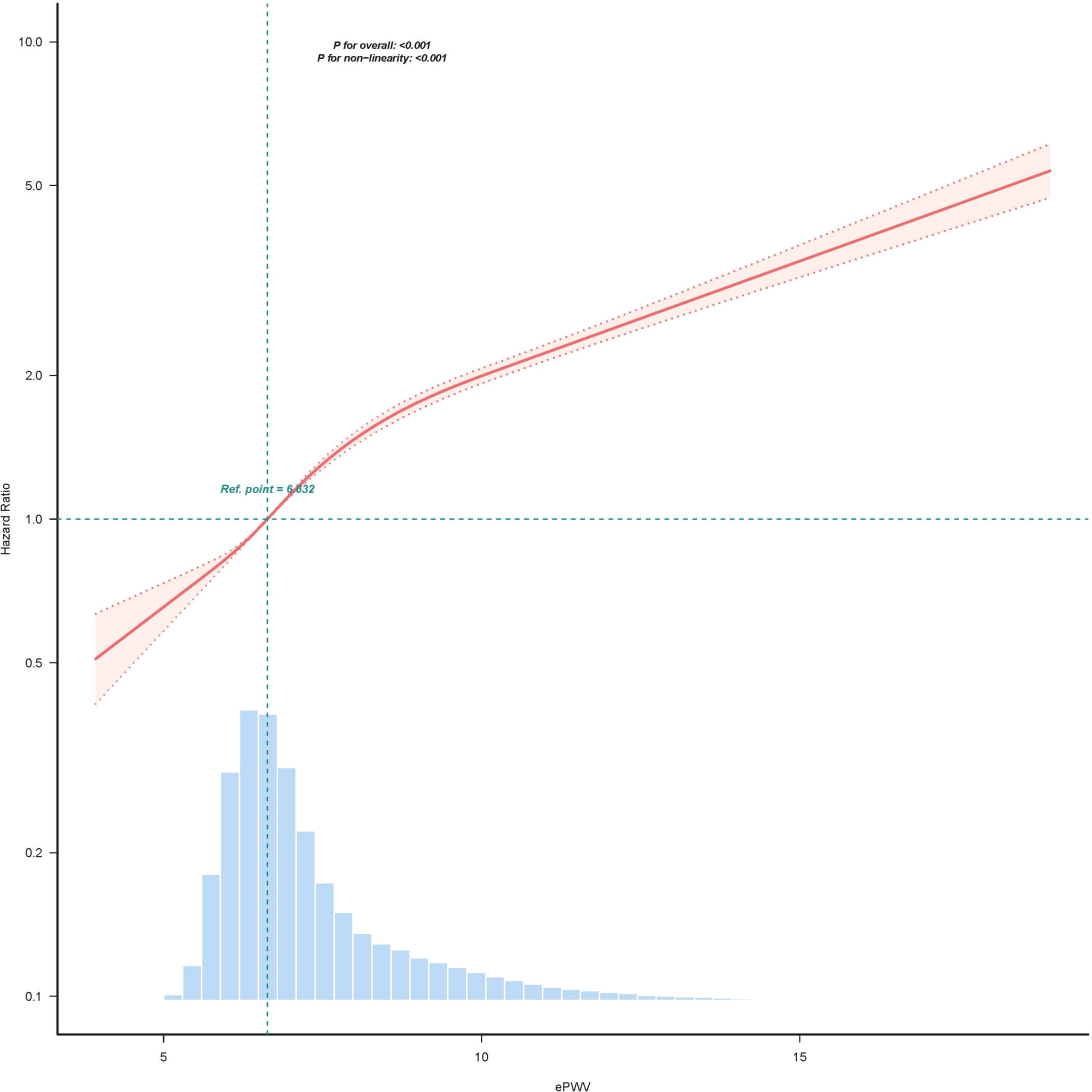
The nonlinear relationship between ePWV and the risk of IFG. We adjusted variables including gender, BMI, FPG, ALT, AST, TG, TC, LDL-C, HDL-C, BUN, Scr, smoking status, and family history of DM. A light blue histogram illustrates the percentage density distribution of participants’ ePWV. A horizontal dashed line denotes a reference HR of 1.0, which serves as the comparative baseline. The estimated adjusted HRs are illustrated by the thick central lines, with shaded regions flanking the curve denoting their respective 95% confidence intervals. A nonlinearity *P*-value below 0.001 signified a non-linear association involving ePWV and the risk of IFG.

Restricted cubic spline analysis revealed a non-linear association between ePWV and the risk of developing IFG (**Figure 5**), with a *P* for nonlinearity < 0.001. **Table 3** presents the threshold analysis. After adjustment for baseline characteristics including gender, BMI, FPG, ALT, AST, TG, TC, LDL−C, HDL−C, BUN, Scr, smoking status, and family history of DM, the inflection point of ePWV was identified at 8.365 m/s. Below 8.365 m/s, each 1 m/s increase in ePWV was associated with a 33% higher risk​ of IFG (HR = 1.33, 95% CI: 1.30 – 1.35). In contrast, above 8.365 m/s, the incremental risk associated with each 1 m/s increase was 12%​ (HR = 1.12, 95% CI: 1.11 – 1.14). Application of the likelihood ratio test yielded a statistically significant *P*-value below 0.001, providing evidence for the presence of a threshold effect.

**Table 3:** Threshold effect of ePWV on the incidence of IFG.

ePWV	HR (95%CI)	*P*-Value
< 8.365	1.33 (1.30, 1.35)	< 0.001
≥ 8.365	1.12 (1.11, 1.14)	< 0.001
*P* for log-likelihood ratio test		< 0.001

The covariates that were adjusted for included age, gender, BMI, baseline FPG, ALT, AST, TG, TC, LDL-C, HDL-C, BUN, Scr, smoking status, and family history of DM were all evaluated. A statistically significant result of the likelihood-ratio test (*P* < 0.001) lent support to the existence of a threshold effect, revealing a non-linear association involving ePWV and both the outcome under investigation and the incidence of IFG, with the inflection point identified at 8.365.

### Subgroup analysis

**Figure 6:**
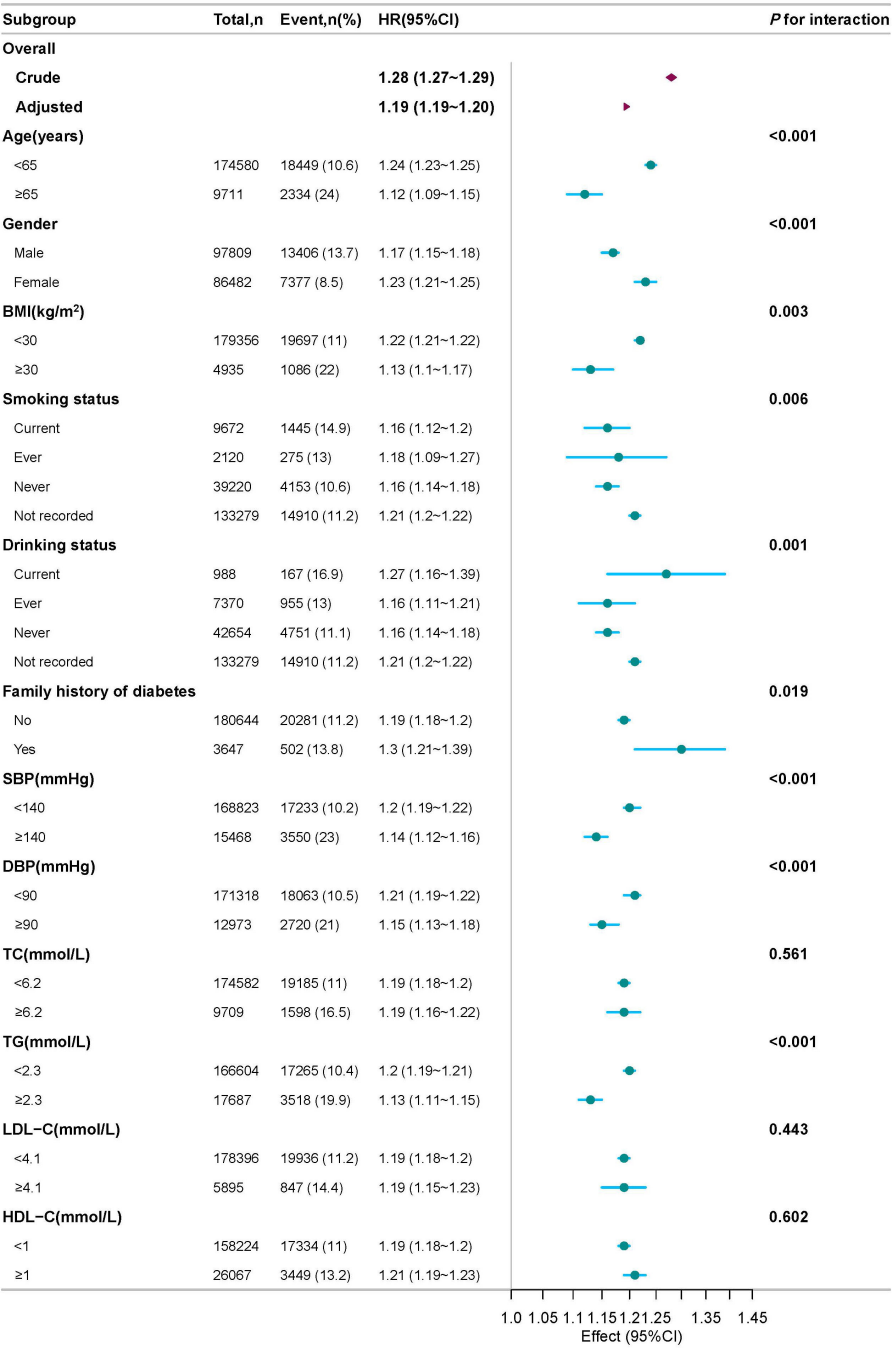
Forest plot of subgroup analysis of the association between ePWV and the risk of IFG.

As illustrated in the forest plot of **Figure 6**, subgroup analyses were performed across prespecified strata, including age, gender, BMI, SBP, DBP, HDL-C, LDL-C, TG, TC, smoking status, drinking status, and family history of DM. Within each subgroup, analyses were adjusted for all baseline covariates except the stratification variable itself, including gender, BMI, FPG, TC, TG, HDL-C, LDL-C, ALT, AST, BUN, Scr, smoking status, drinking status, and family history of DM. In stratified analyses, a positive association (HR > 1) between elevated ePWV and higher IFG risk was observed consistently across all prespecified subgroups, including those defined by age, gender, BMI, blood pressure, and lipid profiles.

### Sensitivity analysis

To assess the robustness of our findings, we performed a series of sensitivity analyses. **Supplementary Table S2** shows that the baseline trends observed in the complete-case dataset (from which all participants with missing variables were excluded) were consistent with those reported in **Table 1**. **Supplementary Table S3** shows that the Cox proportional hazards regression analyses performed on the complete-case dataset produced ePWV-IFG associations largely consistent​ with those reported in **Table 2**. Multivariable Cox regression analyses showed that the positive association between elevated ePWV and an increased risk of IFG remained robust after excluding participants with a baseline BMI ≥ 24 kg/m^2^ (**Supplementary Table S4**). Similarly, the positive association between ePWV and the risk of IFG remained significant and robust in analyses that separately excluded participants aged ≥ 45 years (**Supplementary Table S5**), those with hypertension (**Supplementary Table S6**), or those with hypertension/prehypertension (**Supplementary Table S7**). Finally, a significant positive association remained​ after excluding participants with dyslipidemia (**Supplementary Table S8**). **Supplementary Figure S1** presents the K-M curves for the complete-case dataset and shows the same stepwise increase in cumulative IFG incidence across ePWV quartiles as displayed in **Figure 4**. As shown in the forest plot of **Supplementary Figure S2** using the complete-case dataset, a positive association between elevated ePWV and increased IFG risk was consistently observed across the vast majority of prespecified subgroups, which aligns with the findings of the primary analysis. .

## Discussion

Leveraging multicentre retrospective cohort data from China, this study establishes, to our knowledge, the first international evidence of an independent, positive association between baseline ePWV and incident impaired fasting glucose (IFG). In a fully adjusted model, each 1 m/s increment in ePWV was associated with a 19% increase in IFG risk (HR = 1.19, 95% CI: 1.19 – 1.20). Notably, restricted cubic spline analysis revealed a nonlinear relationship, with a threshold at 8.365 m/s: below this point, IFG risk increased sharply with higher ePWV (HR = 1.33 per 1 m/s), whereas above it, the association attenuated (HR = 1.12 per 1 m/s). This pattern remained consistent across all subgroup and sensitivity analyses. As the first large-scale Chinese cohort to identify ePWV as an independent predictor of IFG, our work not only confirms its role in early dysglycemia but also provides a novel, quantitative threshold that may enhance precision in risk stratification.

The association between elevated ePWV and incident IFG is likely mediated through multiple, interrelated pathophysiological pathways. First, a vascular-to-metabolic causal route may be involved. Elevated ePWV, indicating reduced large-artery elasticity and increased central pulse pressure, alters endothelial shear stress and reduces nitric oxide bioavailability. The resultant microcirculatory hypoperfusion promotes rarefaction of skeletal muscle capillaries, impairs insulin-mediated glucose uptake, and leads to compensatory hyperinsulinemia, ultimately progressing to insulin resistance (24, 25). Second, arterial remodeling—characterized by quantitative and qualitative changes in elastic and collagen fibers—is coupled with chronic low-grade inflammation and oxidative stress. Proinflammatory cytokines such as TNF-α and IL-6 activate the NF-κB signaling pathway, which inhibits tyrosine phosphorylation of IRS-1 and disrupts insulin signal transduction. Simultaneously, reactive oxygen species promote pancreatic β-cell apoptosis via p38 MAPK and JNK pathways, accelerating glycemic deterioration (26). Third, rising glucose levels promote the formation of AGEs, which form cross-links with collagen to directly increase arterial stiffness and bind to RAGE, triggering local pancreatic inflammation and oxidative stress. This creates a positive “vascular-islet” feedback loop. AGEs–RAGE activation upregulates nicotinamide adenine dinucleotide phosphate oxidase, increasing oxidative stress that in turn enhances AGE cross-linking, thereby establishing a pathological cycle between arterial stiffness and hyperglycemia. Moreover, hyperinsulinemia itself may increase arterial stiffness by stimulating vascular smooth muscle cell proliferation, promoting collagen synthesis, and inhibiting matrix metalloproteinase activity, suggesting insulin resistance can further elevate ePWV (27). One study reported that individuals in the highest tertile of carotid–femoral pulse wave velocity had a 3.24-fold higher risk of diabetes compared with the lowest tertile, supporting arterial stiffness as an independent determinant of diabetes risk (28). In summary, ePWV serves not only as an early marker of prediabetes but also appears to drive disease onset and progression via convergent mechanisms involving endothelial dysfunction, inflammatory-oxidative stress, AGE–RAGE interaction, and a positive insulin–vascular feedback loop. The pathophysiological pathways delineated above, while supported by prior experimental and clinical studies, remain hypothetical in the context of our present findings. It is important to note that the original dataset did not include measurements of​ key intermediary biomarkers such as endothelial function (e.g., flow-mediated dilation), inflammatory cytokines (e.g., TNF-α and IL-6), oxidative stress markers, or AGEs. Therefore, the proposed mechanistic links—from increased ePWV to endothelial dysfunction, inflammation, and subsequently to impaired insulin signaling and β-cell function—represent a plausible biological framework​ derived from the existing literature rather than causally proven pathways within our cohort. Future studies incorporating these direct measurements are necessary to validate these mechanisms. Consequently, At both the clinical and investigative levels, incorporating ePWV into pre-DM risk-stratification models is expected to furnish a novel therapeutic target and an evidence-based rationale for interventions designed to arrest or reverse the progression of hyperglycaemia.

Our results align with numerous investigations that have investigated the association between vascular function and glucose metabolism abnormalities. In a Chinese cohort, Wu et al. demonstrated that MAP independently predicted five-year incident DM, with a nonlinear dose-response pattern. When MAP was below 100.333 mmHg, a strong positive relationship between MAP and DM was identified (29). Likewise, Lin et al. documented a curvilinear link between MAP and the occurrence of IFG, underscoring the potential pathophysiological interplay between arterial pressure and early glucose metabolism alterations (30). Dong et al. demonstrated that increased ePWV and BMI serve as independent predictors of DM. Combining the two indices yields superior predictive accuracy for new-onset DM than either measure alone (20). The investigation by Liu et al. revealed a linear association between the TyG index and the onset of incident IFG among older Chinese adults, allowing clinicians to recognize individuals at elevated risk and apply focused interventions to lower their probability of advancing to DM (31). In a Chinese cohort, Cao et al. documented a significant positive correlation between pulse pressure (PP) and the risk of pre-DM (HR = 1.009, 95%CI = 1.007 – 1.010), implying that arterial stiffening may serve as an early harbinger of DM development (32). Wen et al. described a strong link between the TyG index and the onset of pre-DM (HR = 2.1, 95%CI = 2.0 – 2.1), reinforcing the central role of metabolic vascular injury in the pathogenesis of glucose dysregulation (33). The novelty of the present study lies in being the first to link the readily obtainable arterial stiffness marker ePWV with incident IFG and to identify a non-linear inflection point at 8.365 m/s. This observation echoes two related reports: the investigation by Pan et al. regarding the association between pulse pressure index (PPI) and the risk of pre-DM (34), and Cao et al.’s illustration of a curvilinear association between PP and pre-DM risk (32). These studies consistently indicate that the relationship between vascular parameters and glucose dysregulation may exhibit threshold effects. Consistent with the broader literature, our nonlinear model delineates the ePWV risk gradient with greater precision, revealing a sharp rise in IFG risk once ePWV falls below 8.365 m/s. This inflection point furnishes a refined threshold that can guide earlier and more targeted intervention in high-risk individuals.

Our findings carry direct clinical and public-health relevance. Being non-invasive, inexpensive, and readily obtainable in routine check-ups, ePWV may serve as a potential​ early screening tool for pre-DM, particularly enabling primary-care facilities to flag and risk-stratify high-risk individuals (35, 36). However, it is crucial to interpret this implication with caution.​ Our study quantified the strength of association (hazard ratios) but did not evaluate the predictive performance​ of ePWV for incident IFG, such as its discriminative accuracy (e.g., C-statistic, ROC analysis) or calibration. Therefore, the utility of ePWV as a standalone or integrated predictive marker requires formal assessment in future studies specifically designed for prediction model development and validation. Early lifestyle interventions for individuals identified as high-risk by such future, validated models​ may significantly delay or prevent the onset of DM, thereby achieving primary prevention (37, 38). Future studies should examine whether incorporating ePWV into current DM risk prediction models enhances predictive accuracy and clinical applicability further.

The chief advantage of this investigation lies in its large sample size and multicentre longitudinal design. Data from 184,291 adults recruited across 32 centres in 11 Chinese cities provide a representative sample and enhance the credibility of the findings. A mean follow-up of more than three years offers robust temporal evidence for the link involving ePWV and the development of incident IFG. In addition, a wide spectrum of potential confounders, including lipid profile, liver function indices, and lifestyle factors, was rigorously controlled through multivariable Cox proportional-hazards modelling, which substantially strengthens the robustness of the results. Most importantly, this is the first study in a Chinese population to delineate a curvilinear relationship association involving ePWV and the likelihood of IFG, to quantify the inflection point at 8.365 m/s, and to provide a refined threshold for risk stratification of high-risk individuals. Restricted cubic spline analysis and extensive subgroup examinations corroborated both the stability and heterogeneity of the ePWV-IFG association, underscoring the methodological rigor of the present investigation.

Several caveats merit consideration in the interpretation of our results. First, the retrospective, observational design cannot fully eliminate residual confounding by unmeasured factors such as post-load glucose, glycated haemoglobin, dietary patterns, physical-activity levels, or socioeconomic status. The observed associations therefore do not imply causality. Second, ePWV was estimated from age and BP rather than being measured directly by the reference-standard carotid–femoral pulse wave velocity, so random measurement error is possible, although the validity of this estimate has been supported by previous work. Third, because the cohort was drawn from Chinese adults attending routine health check-ups, the participants were probably healthier than the general community population and were ethnically homogeneous. Consequently, extrapolation of the findings to other racial groups or to broader community settings outside China should be undertaken cautiously. Fourth, information on family history of DM and some other variables was collected by self-reported data, which is prone to recall bias. Fifth, the analysis used a pre-processed dataset derived from an earlier survey. Because only the processed files were available, we were unable to evaluate potential selection bias introduced when participants were excluded and drawn from the initial cohort (n = 685, 277) to generate the harmonized dataset (n = 211, 833). Sixth, as a secondary analysis of retrospective health screening data, the intervals between FPG measurements were variable and not prescribed by a study protocol. Moreover, incident IFG was defined based on a single FPG measurement meeting the ADA criterion, without confirmation by a repeat test, which is a common operational approach in large-scale epidemiological studies for risk stratification. Seventh, a critical methodological limitation is that the proportional hazards assumption underlying the Cox regression models was not formally tested (e.g., using Schoenfeld residuals).​ Eighth, and importantly, the observational design cannot determine the direction of the observed association.​ While we hypothesize that increased arterial stiffness (higher ePWV) contributes to the development of IFG, the possibility of reverse causality—whereby early glucose metabolic abnormalities lead to increased arterial stiffness—cannot be ruled out. This bidirectional relationship could form a vicious cycle, which our retrospective data are unable to disentangle. While the models provided estimates of the average hazard ratio over the follow-up period, violation of this assumption could affect the reliability of the HR estimates. Our findings regarding the association between ePWV and IFG risk should therefore be interpreted with this caveat in mind. Notwithstanding these constraints, the investigation retains considerable merit, as it provides an in-depth examination of a rigorously characterized, large-scale health-screening cohort, thus furnishing distinctive perspectives on the research issue. We will address the identified limitations and refine evidence on the ePWV-IFG link through a multicentre prospective design that, through several key measures: (1) Collecting prospective data on post-load glucose, HbA1c, dietary intake, and objectively measured physical activity to minimize residual confounding and strengthen causal inference; (2) Acquiring concurrent carotid-femoral pulse-wave velocity measurements to quantify and correct estimation errors where feasible; (3) Enrolling multi-ethnic, community-based cohorts to evaluate external validity across diverse populations; (4) Validating family history using biomarkers or registry records to reduce recall bias; and (5) Extending follow-up to at least five years to enhance statistical power and fully characterize the temporal relationship between ePWV and incident IFG.

## Conclusions

In this extensive multicenter retrospective cohort investigation encompassing 184,291 Chinese adults, we demonstrated that elevated ePWV is independently linked to a heightened risk of incident IFG, even following thorough adjustment for demographic, clinical, and metabolic confounding factors. The results indicate a notable positive association between ePWV and IFG incidence, with each 1 m/s increment in ePWV corresponding to a 19% elevated risk of IFG. Importantly, we observed a non-linear link between ePWV and the likelihood of IFG, featuring a turning point at 8.365 m/s. At values lower than this cutoff, the association was more pronounced (HR = 1.33), while above it, the risk increase attenuated (HR = 1.12). Subgroup analyses confirmed the consistency of this relationship across diverse population strata, including age, gender, BMI, blood pressure, lipid profiles, lifestyle factors, and family history of DM. These results underscore the possible utility of ePWV as a simple, non-invasive indicator for early detection of persons at elevated risk of IFG, which may facilitate targeted interventions to prevent DM progression. However, given the retrospective design and owing to the employment of estimated rather than directly assessed PWV, additional prospective investigations are required to verify causal relationships and elucidate underlying mechanisms.

## Data Availability

Publicly available datasets were analyzed in this study. This data can be found here: The complete dataset supporting these analyses is openly retrievable from the Dryad repository at https://doi.org/10.5061/dryad.ft8750v.
