# Supplementary material for: Association between estimated pulse wave velocity and impaired fasting glucose: a multicenter retrospective cohort study in China
Source: Front Endocrinol (Lausanne). 2026 Apr 27;17:1763973. doi: 10.3389/fendo.2026.1763973 (PMC13158118; doi:10.3389/fendo.2026.1763973)
Supplement: Supplementary file 1 [file SupplementaryFile1.docx]

**Supplementary Table 1 Description of missing data.**

| **Variables** | **Non-missing** | **Missing** | **Miss percentage(%)** |
| --- | --- | --- | --- |
| Age | 184291 | 0 | 0 |
| Gender | 184291 | 0 | 0 |
| BMI | 184291 | 0 | 0 |
| ePWV | 184291 | 0 | 0 |
| SBP | 184291 | 0 | 0 |
| DBP | 184291 | 0 | 0 |
| MAP | 184291 | 0 | 0 |
| FPG | 184291 | 0 | 0 |
| TC | 184291 | 4213 | 2.2861 |
| TG | 180047 | 4244 | 2.3029 |
| HDL-C | 100890 | 83401 | 45.2551 |
| LDL-C | 101393 | 82898 | 44.9821 |
| ALT | 182750 | 1541 | 0.8362 |
| AST | 76582 | 107709 | 58.4451 |
| BUN | 165721 | 18570 | 10.0765 |
| Scr | 174525 | 9766 | 5.2992 |
| Smoking status | 51012 | 133279 | 72.3199 |
| Drinking status | 51012 | 133279 | 72.3199 |
| Family history of DM | 184291 | 0 | 0 |

**Supplementary Table 2** **Baseline characteristics of the study population after excluding participants with incomplete data of covariates.**

| Variables | Total  (n = 10471) | ePWV | | | | |
| --- | --- | --- | --- | --- | --- | --- |
|  |  | Q1  (n = 2616) | Q2  (n = 2613) | Q3  (n = 2624) | Q4  (n = 2618) | *P*-Value |
| ePWV (m/s), Mean ± SD | 7.08 ± 1.38 | 5.81 ± 0.25 | 6.42 ± 0.16 | 7.06 ± 0.24 | 9.02 ± 1.27 | < 0.001 |
| Age (years), Mean ± SD | 40.88 ± 11.11 | 33.93 ± 5.10 | 35.13 ± 6.08 | 39.60 ± 7.64 | 54.82 ± 9.76 | < 0.001 |
| Gender, n (%) |  |  |  |  |  | < 0.001 |
| Male | 6859 (65.50) | 1169 (44.69) | 1805 (69.08) | 2006 (76.45) | 1879 (71.77) |  |
| Female | 3612 (34.50) | 1447 (55.31) | 808 (30.92) | 618 (23.55) | 739 (28.23) |  |
| Height (cm),  Mean ± SD | 167.72 ± 8.12 | 165.75 ± 8.21 | 168.89 ± 8.05 | 169.40 ± 7.62 | 166.83 ± 8.06 | < 0.001 |
| Body weight (kg), Mean ± SD | 66.01 ± 12.01 | 60.06 ± 10.76 | 66.12 ± 11.95 | 69.12 ± 11.94 | 68.74 ± 11.11 | < 0.001 |
| BMI (kg/m^2^), Mean ± SD | 23.36 ± 3.24 | 21.75 ± 2.79 | 23.07 ± 3.15 | 23.99 ± 3.25 | 24.62 ± 3.01 | < 0.001 |
| SBP (mmHg), Mean ± SD | 117.98 ± 14.93 | 103.76 ± 8.31 | 115.48 ± 9.33 | 122.26 ± 11.52 | 130.41 ± 14.87 | < 0.001 |
| DBP (mmHg), Mean ± SD | 74.12 ± 10.27 | 63.42 ± 4.99 | 71.97 ± 5.04 | 78.32 ± 7.48 | 82.76 ± 10.22 | < 0.001 |
| MAP (mmHg), Mean ± SD | 88.74 ± 10.90 | 76.87 ± 4.73 | 86.47 ± 4.67 | 92.96 ± 7.58 | 98.64 ± 10.56 | < 0.001 |
| Baseline FPG (mmol/L), Mean ± SD | 4.84 ± 0.46 | 4.76 ± 0.47 | 4.81 ± 0.46 | 4.87 ± 0.46 | 4.92 ± 0.45 | < 0.001 |
| TC (mmol/L), Mean ± SD | 4.72 ± 0.88 | 4.46 ± 0.79 | 4.61 ± 0.83 | 4.80 ± 0.87 | 5.02 ± 0.90 | < 0.001 |
| TG (mmol/L), M (IQR) | 1.11 (0.77, 1.68) | 0.86 (0.61, 1.20) | 1.05 (0.74, 1.58) | 1.23 (0.85, 1.85) | 1.40 (1.00, 2.08) | < 0.001 |
| HDL-C (mmol/L), Mean ± SD | 1.35 ± 0.29 | 1.39 ± 0.29 | 1.35 ± 0.28 | 1.33 ± 0.29 | 1.33 ± 0.29 | < 0.001 |
| LDL-C (mmol/L), Mean ± SD | 2.71 ± 0.68 | 2.51 ± 0.61 | 2.63 ± 0.65 | 2.77 ± 0.66 | 2.93 ± 0.70 | < 0.001 |
| ALT (U/L), M (IQR) | 19.30 (13.50, 29.00) | 15.00 (11.10, 22.00) | 19.30 (13.20, 30.00) | 22.50 (15.50, 34.00) | 21.40 (15.70, 30.10) | < 0.001 |
| AST (U/L), M (IQR) | 22.60 (19.00, 27.30) | 20.80 (17.90, 24.70) | 22.10 (19.00, 27.00) | 23.20 (19.80, 28.80) | 24.00 (20.50, 29.00) | < 0.001 |
| BUN (mmol/L), Mean ± SD | 4.70 ± 1.16 | 4.44 ± 1.11 | 4.61 ± 1.14 | 4.72 ± 1.13 | 5.01 ± 1.19 | < 0.001 |
| Scr (μmol/L), Mean ± SD | 73.35 ± 15.24 | 68.39 ± 15.15 | 74.15 ± 14.96 | 75.99 ± 14.61 | 74.86 ± 15.11 | < 0.001 |
| Smoking status, n (%) |  |  |  |  |  | < 0.001 |
| Current smoker | 1999 (19.09) | 287 (10.97) | 414 (15.84) | 523 (19.93) | 775 (29.6) |  |
| Ever smoker | 454 ( 4.34) | 85 (3.25) | 117 (4.48) | 143 (5.45) | 109 (4.16) |  |
| Never smoker | 8018 (76.57) | 2244 (85.78) | 2082 (79.68) | 1958 (74.62) | 1734 (66.23) |  |
| Drinking status, n (%) |  |  |  |  |  | < 0.001 |
| Current drinker | 282 ( 2.69) | 22 (0.84) | 45 (1.72) | 66 (2.52) | 149 (5.69) |  |
| Ever drinker | 2065 (19.72) | 373 (14.26) | 563 (21.55) | 639 (24.35) | 490 (18.72) |  |
| Never drinker | 8124 (77.59) | 2221 (84.9) | 2005 (76.73) | 1919 (73.13) | 1979 (75.59) |  |
| Family history of DM, n (%) |  |  |  |  |  | 0.153 |
| No | 9851 (94.08) | 2471 (94.46) | 2462 (94.22) | 2445 (93.18) | 2473 (94.46) |  |
| Yes | 620 ( 5.92) | 145 (5.54) | 151 (5.78) | 179 (6.82) | 145 (5.54) |  |
| IFG, n (%) |  |  |  |  |  | < 0.001 |
| No | 9211 (87.97) | 2450 (93.65) | 2406 (92.08) | 2271 (86.55) | 2084 (79.6) |  |
| Yes | 1260 (12.03) | 166 (6.35) | 207 (7.92) | 353 (13.45) | 534 (20.4) |  |

**Supplementary Table 3 The association between ePWV and the risk of IFG in different**

**models** **among the study population after excluding participants with incomplete data of covariates (n = 10471).**

| Variables | Crude model | | Model Ⅰ | | Model Ⅱ | |
| --- | --- | --- | --- | --- | --- | --- |
|  | HR (95%CI) | *P*-Value | HR (95%CI) | *P*-Value | HR (95%CI) | *P*-Value |
| ePWV, m/s | 1.27 (1.23, 1.31) | < 0.001 | 1.19 (1.18, 1.20) | < 0.001 | 1.15 (1.11, 1.19) | < 0.001 |
| (ePWV quartiles) |  |  |  |  |  |  |
| Q1 | 1. (Reference) |  | 1.00 (Reference) |  | 1.00 (Reference) |  |
| Q2 | 1.2 (0.98, 1.48) | 0.076 | 0.95 (0.77, 1.17) | 0.615 | 0.94 (0.76, 1.15) | 0.539 |
| Q3 | 2.01 (1.68, 2.42) | < 0.001 | 1.3 (1.08, 1.58) | 0.007 | 1.26(1.04, 1.53) | 0.019 |
| Q4 | 3.05 (2.57, 3.63) | < 0.001 | 1.77 (1.47, 2.12) | < 0.001 | 1.68(1.39, 2.02) | < 0.001 |
| *P* for trend |  | < 0.001 |  | < 0.001 |  | < 0.001 |

Crude model: we did not adjust other covariates.
Model I: adjusted for gender, BMI, and FPG at baseline.

Model II: further adjusted for TC, TG, HDL-C, LDL-C, ALT, AST, BUN, Scr, smoking status, drinking status, and family history of DM.

**Supplementary Table 4** **The association between ePWV and the risk of IFG in different models based on unimputed data, after excluding participants with BMI ≥ 24 kg/m^2^ (n = 117747).**

| Variables | Crude model | | Model Ⅰ | | Model Ⅱ | |
| --- | --- | --- | --- | --- | --- | --- |
|  | HR (95%CI) | *P*-Value | HR (95%CI) | *P*-Value | HR (95%CI) | *P*-Value |
| ePWV, m/s | 1.31 (1.29, 1.32) | < 0.001 | 1.22 (1.21, 1.23) | < 0.001 | 1.23 (1.21, 1.25) | < 0.001 |
| (ePWV quartiles) |  |  |  |  |  |  |
| Q1 | 1.00 (Reference) |  | 1.00 (Reference) |  | 1.00 (Reference) |  |
| Q2 | 1.32 (1.23, 1.42) | < 0.001 | 1.11 (1.03, 1.2) | 0.005 | 1.12 (0.96, 1.31) | 0.139 |
| Q3 | 1.72 (1.61, 1.84) | < 0.001 | 1.28 (1.19, 1.37) | < 0.001 | 1.48 (1.28, 1.7) | < 0.001 |
| Q4 | 3.45 (3.24, 3.67) | < 0.001 | 2.18 (2.04, 2.32) | < 0.001 | 2.6 (2.28, 2.97) | < 0.001 |
| *P* for trend |  | < 0.001 |  | < 0.001 |  | < 0.001 |

Crude model: we did not adjust other covariates.
Model I: adjusted for gender, BMI, and FPG at baseline.

Model II: further adjusted for TC, TG, HDL-C, LDL-C, ALT, AST, BUN, Scr, smoking status, drinking status, and family history of DM.

**Supplementary Table 5** **The association between ePWV and the risk of IFG in different models based on unimputed data, after excluding participants aged aged ≥ 45 years (n = 125379).**

| Variables | Crude model | | Model Ⅰ | | Model Ⅱ | |
| --- | --- | --- | --- | --- | --- | --- |
|  | HR (95%CI) | *P*-Value | HR (95%CI) | *P*-Value | HR (95%CI) | *P*-Value |
| ePWV, m/s | 1.67 (1.62, 1.71) | < 0.001 | 1.25 (1.21, 1.28) | < 0.001 | 1.26 (1.18, 1.33) | < 0.001 |
| (ePWV quartiles) |  |  |  |  |  |  |
| Q1 | 1.00 (Reference) |  | 1.00 (Reference) |  | 1.00 (Reference) |  |
| Q2 | 1.37 (1.28, 1.46) | < 0.001 | 1.09 (1.02, 1.17) | 0.009 | 1.11 (0.97, 1.27) | 0.142 |
| Q3 | 1.69 (1.59, 1.8) | < 0.001 | 1.16 (1.08, 1.23) | < 0.001 | 1.17 (1.02, 1.33) | 0.023 |
| Q4 | 2.56 (2.41, 2.71) | < 0.001 | 1.42 (1.33, 1.51) | < 0.001 | 1.44 (1.26, 1.64) | < 0.001 |
| *P* for trend |  | < 0.001 |  | < 0.001 |  | < 0.001 |

Crude model: we did not adjust other covariates.
Model I: adjusted for gender, BMI, and FPG at baseline.

Model II: further adjusted for TC, TG, HDL-C, LDL-C, ALT, AST, BUN, Scr, smoking status, drinking status, and family history of DM.

**Supplementary Table 6 The association between ePWV and the risk of IFG in different models based on unimputed data, after excluding participants with hypertension (n = 162427).**

| Variables | Crude model | | Model Ⅰ | | Model Ⅱ | |
| --- | --- | --- | --- | --- | --- | --- |
|  | HR (95%CI) | *P*-Value | HR (95%CI) | *P*-Value | HR (95%CI) | *P*-Value |
| ePWV, m/s | 1.32 (1.31, 1.34) | < 0.001 | 1.21 (1.19, 1.22) | < 0.001 | 1.25 (1.23, 1.28) | < 0.001 |
| (ePWV quartiles) |  |  |  |  |  |  |
| Q1 | 1.00 (Reference) |  | 1.00 (Reference) |  | 1.00 (Reference) |  |
| Q2 | 1.36 (1.29, 1.44) | < 0.001 | 1.1 (1.04, 1.17) | 0.001 | 1.14 (1.01, 1.28) | 0.027 |
| Q3 | 1.83 (1.74, 1.93) | < 0.001 | 1.26 (1.2, 1.33) | < 0.001 | 1.32 (1.19, 1.48) | < 0.001 |
| Q4 | 3.15 (3, 3.31) | < 0.001 | 1.87 (1.78, 1.97) | < 0.001 | 2.11 (1.9, 2.34) | < 0.001 |
| *P* for trend |  | < 0.001 |  | < 0.001 |  | < 0.001 |

Crude model: we did not adjust other covariates.
Model I: adjusted for gender, BMI, and FPG at baseline.

Model II: further adjusted for TC, TG, HDL-C, LDL-C, ALT, AST, BUN, Scr, smoking status, drinking status, and family history of DM.

**Supplementary Table 7 The association between ePWV and the risk of IFG in different models based on unimputed data, after excluding participants with hypertension or prehypertension (n = 97032).**

| Variables | Crude model | | Model Ⅰ | | Model Ⅱ | |
| --- | --- | --- | --- | --- | --- | --- |
|  | HR (95%CI) | *P*-Value | HR (95%CI) | *P*-Value | HR (95%CI) | *P*-Value |
| ePWV, m/s | 1.4 (1.37, 1.42) | < 0.001 | 1.23 (1.2, 1.25) | < 0.001 | 1.27 (1.22, 1.31) | < 0.001 |
| (ePWV quartiles) |  |  |  |  |  |  |
| Q1 | 1.00 (Reference) |  | 1.00 (Reference) |  | 1.00 (Reference) |  |
| Q2 | 1.37 (1.27, 1.49) | < 0.001 | 1.17 (1.09, 1.27) | < 0.001 | 1.16 (0.99, 1.36) | 0.072 |
| Q3 | 1.54 (1.42, 1.66) | < 0.001 | 1.15 (1.07, 1.25) | < 0.001 | 1.18 (1.01, 1.38) | 0.039 |
| Q4 | 2.64 (2.46, 2.83) | < 0.001 | 1.63 (1.52, 1.75) | < 0.001 | 1.75 (1.51, 2.02) | < 0.001 |
| *P* for trend |  | < 0.001 |  | < 0.001 |  | < 0.001 |

Crude model: we did not adjust other covariates.
Model I: adjusted for gender, BMI, and FPG at baseline.

Model II: further adjusted for TC, TG, HDL-C, LDL-C, ALT, AST, BUN, Scr, smoking status, drinking status, and family history of DM.

**Supplementary Table 8 The association between ePWV and the risk of IFG in different models based on unimputed data, after excluding participants with dyslipidemia (n = 73764).**

| Variables | Crude model | | Model Ⅰ | | Model Ⅱ | |
| --- | --- | --- | --- | --- | --- | --- |
|  | HR (95%CI) | *P*-Value | HR (95%CI) | *P*-Value | HR (95%CI) | *P*-Value |
| ePWV, m/s | 1.28 (1.26, 1.29) | < 0.001 | 1.18 (1.16, 1.19) | <0.001 | 1.22 (1.2, 1.24) | < 0.001 |
| (ePWV quartiles) |  |  |  |  |  |  |
| Q1 | 1.00 (Reference) |  | 1.00 (Reference) |  | 1.00 (Reference) |  |
| Q2 | 1.41 (1.3, 1.54) | < 0.001 | 1.15 (1.05, 1.25) | 0.001 | 1.26 (1.12, 1.43) | < 0.001 |
| Q3 | 2.08 (1.92, 2.25) | < 0.001 | 1.42 (1.31, 1.54) | < 0.001 | 1.6 (1.42, 1.8) | < 0.001 |
| Q4 | 3.9 (3.63, 4.2) | < 0.001 | 2.22 (2.06, 2.4) | < 0.001 | 2.71 (2.42, 3.04) | < 0.001 |
| *P* for trend |  | < 0.001 |  | < 0.001 |  | < 0.001 |

Crude model: we did not adjust other covariates.
Model I: adjusted for gender, BMI, and FPG at baseline.

Model II: further adjusted for TC, TG, HDL-C, LDL-C, ALT, AST, BUN, Scr, smoking status, drinking status, and family history of DM.

**Supplementary Figure 1 K-M curves illustrate IFG incidence by ePWV quartiles among**

**the study population after excluding participants with incomplete data of covariates (Log-rank test *P* < 0.0001).**


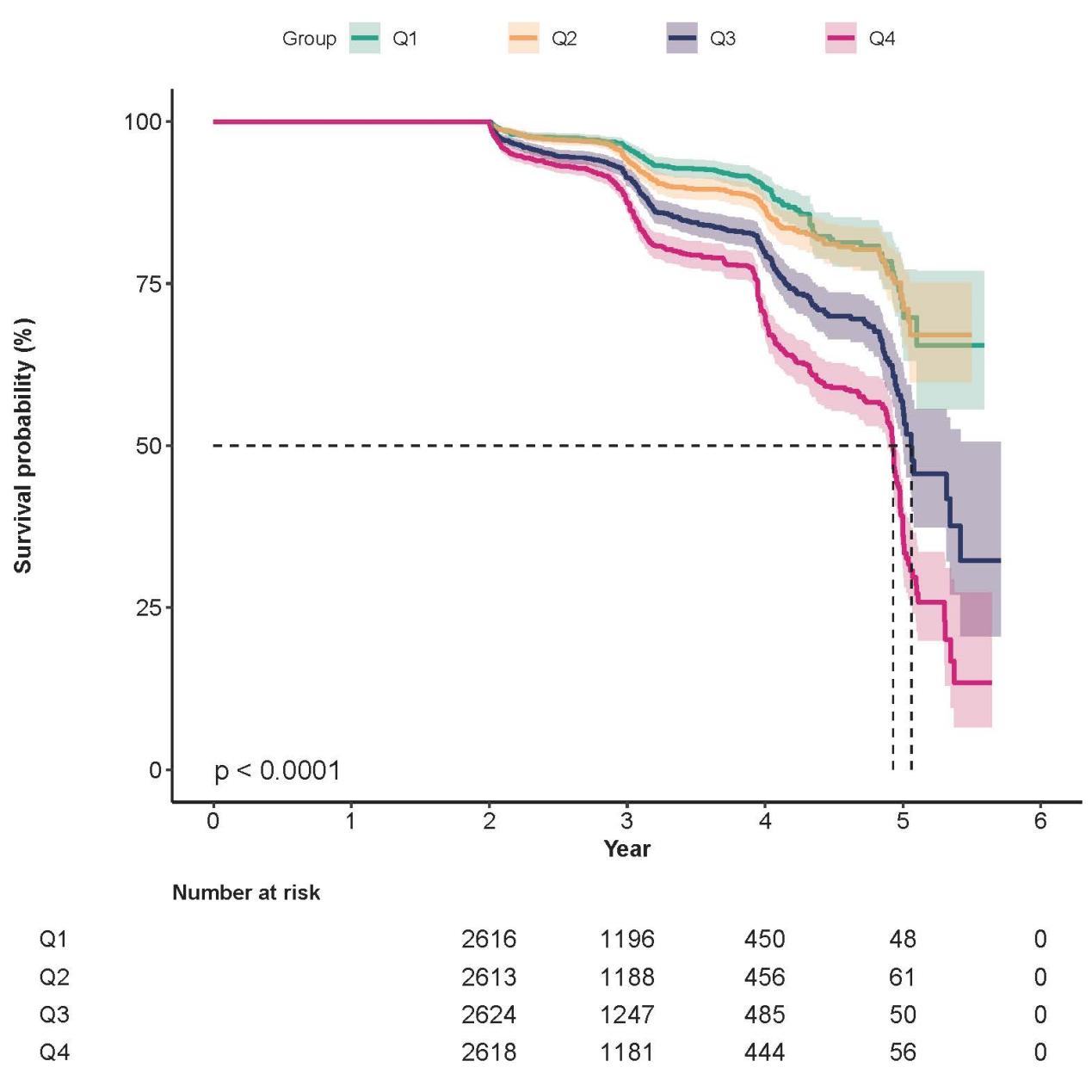


**Supplementary Figure 2** **Forest plot of subgroup analysis of the association between ePWV and the risk of IFG among the study population after excluding participants with incomplete data of covariates.** As illustrated in the figure, subgroup analyses were conducted across prespecified strata, including age, gender, BMI, SBP, DBP, HDL-C, LDL-C, TG, TC, smoking status, drinking status, and family history of diabetes. Within each subgroup, analyses were adjusted for all baseline covariates except the stratification variable itself, including gender, BMI, FPG, TC, TG, HDL-C, LDL-C, ALT, AST, BUN, Scr, smoking status, drinking status, and family history of diabetes. A positive association between elevated ePWV and an increased risk of incident IFG (HR > 1) was consistently observed in the vast majority of these subgroups.
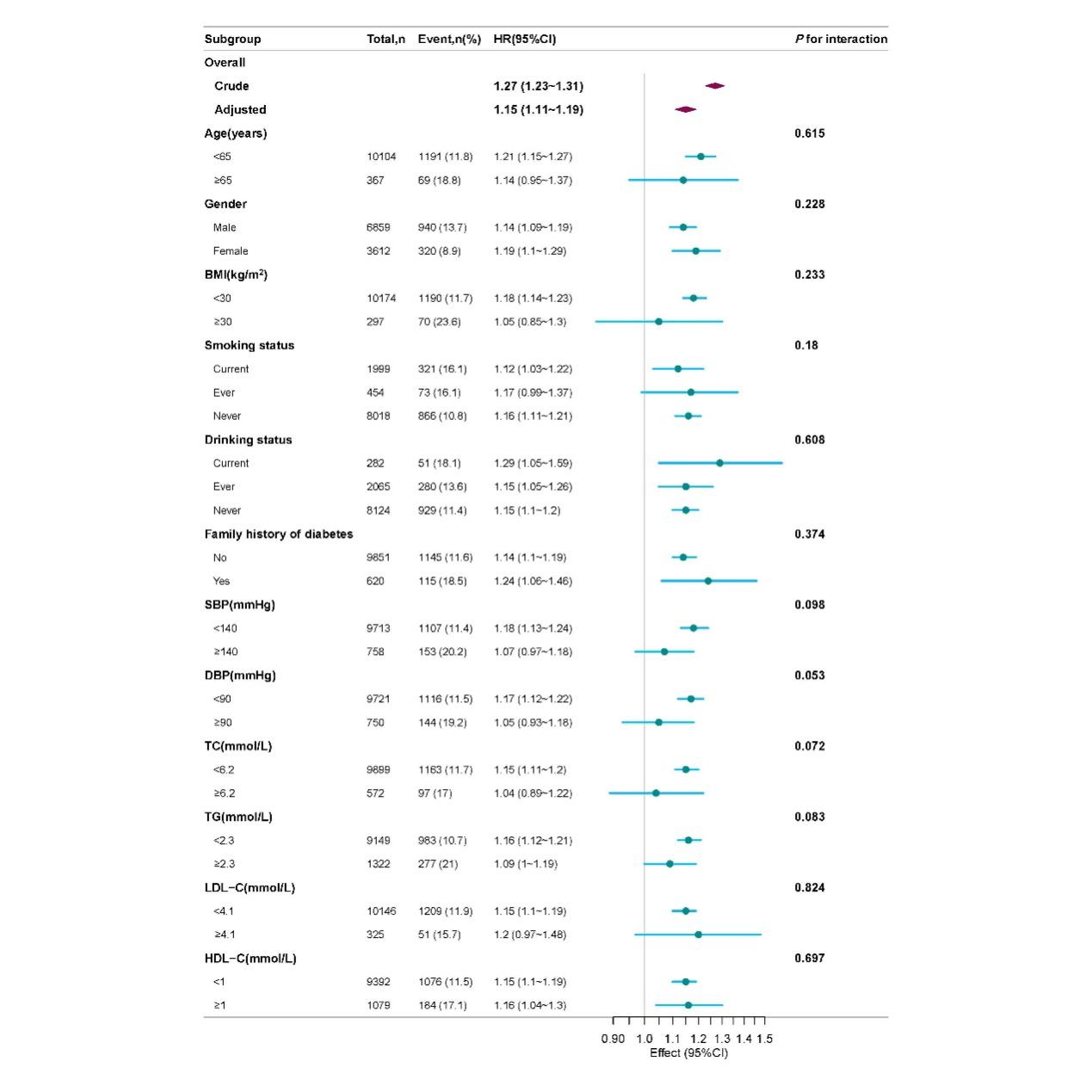


**Supplementary Table 9 Variance inflation factors of covariates included in the multivariable-adjusted model (Model II).**

| **Variables** | **VIF** |
| --- | --- |
| Gender | 1.189 |
| BMI | 1.147 |
| FPG | 1.023 |
| TC | 2.348 |
| TG | 1.204 |
| HDL-C | 1.187 |
| LDL-C | 2.145 |
| ALT | 2.292 |
| AST | 2.208 |
| BUN | 1.194 |
| Scr | 1.263 |
| Smoking status | 1.019 |
| Drinking status | 1.019 |
| Family history of DM | 1.027 |
